# ChemTables: a dataset for semantic classification on tables in chemical patents

**DOI:** 10.1186/s13321-021-00568-2

**Published:** 2021-12-11

**Authors:** Zenan Zhai, Christian Druckenbrodt, Camilo Thorne, Saber A. Akhondi, Dat Quoc Nguyen, Trevor Cohn, Karin Verspoor

**Affiliations:** 1 School of Computing and Information Systems, The University of Melbourne, Melbourne, Australia; 2 Elsevier-Data Science, Life Science, Amsterdam, The Netherlands; 3 VinAI Research, Hanoi, Vietnam; 4 Present Address: School of Computing Technologies, RMIT University, Melbourne, Australia

**Keywords:** Neural networks, Table classification, Chemical patents

## Abstract

Chemical patents are a commonly used channel for disclosing novel compounds and reactions, and hence represent important resources for chemical and pharmaceutical research. Key chemical data in patents is often presented in tables. Both the number and the size of tables can be very large in patent documents. In addition, various types of information can be presented in tables in patents, including spectroscopic and physical data, or pharmacological use and effects of chemicals. Since images of Markush structures and merged cells are commonly used in these tables, their structure also shows substantial variation. This heterogeneity in content and structure of tables in chemical patents makes relevant information difficult to find. We therefore propose a new text mining task of automatically categorising tables in chemical patents based on their contents. Categorisation of tables based on the nature of their content can help to identify tables containing key information, improving the accessibility of information in patents that is highly relevant for new inventions. For developing and evaluating methods for the table classification task, we developed a new dataset, called ChemTables, which consists of 788 chemical patent tables with labels of their content type. We introduce this data set in detail. We further establish strong baselines for the table classification task in chemical patents by applying state-of-the-art neural network models developed for natural language processing, including TabNet, ResNet and Table-BERT on ChemTables. The best performing model, Table-BERT, achieves a performance of 88.66 micro-averaged $F_1$ score on the table classification task. The ChemTables dataset is publicly available at https://doi.org/10.17632/g7tjh7tbrj.3, subject to the CC BY NC 3.0 license. Code/models evaluated in this work are in a Github repository https://github.com/zenanz/ChemTables.

## Introduction

A large number of chemical compounds are first published in patents. It takes on average one to three years for compounds disclosed in patents to appear in scientific literature [1], and only a small fraction of these compounds ever appear at all in publications. Therefore, chemical patents are an important resource for the development of information management tools to support chemical research. Information in patents is crucial for novelty or fact checking and understanding compound prior art [2].

**Fig. 1:**
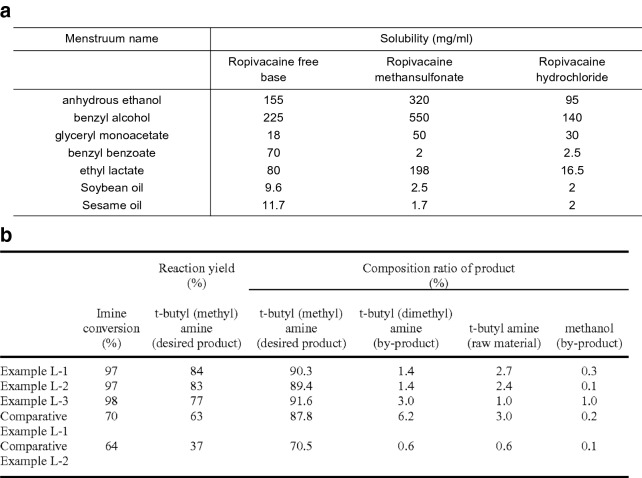
Example of tables in chemical patents that are of high interest to researchers. **a** Example of table which contains solubility data of compounds (*EP2949316A1 Table* 2). **b** Example of table which shows reaction related data. (*US09194041B2 Table* 2)

**Fig. 2:**
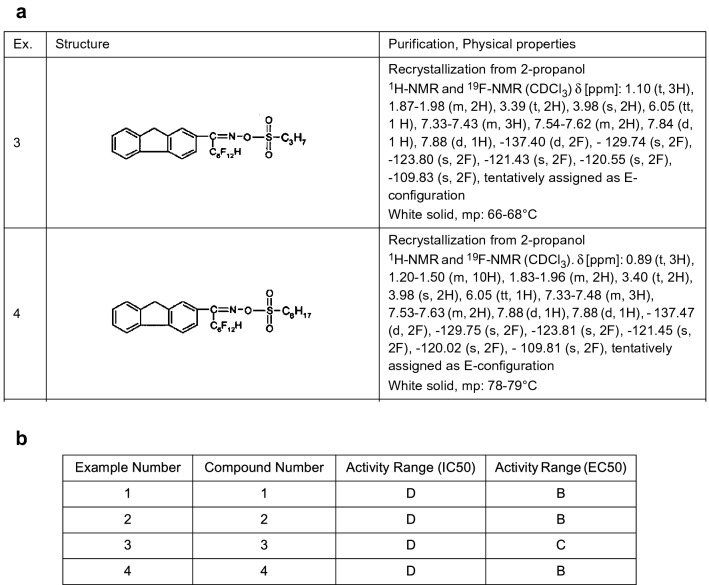
Examples of showing the heterogeneity of tables in chemical patent documents. **a** Example of a self-contained table describing spectroscopic data of compounds. Columns in this table are organized by data format (i.e. images, texts) (*EP1769286B1 Table* 1). **b** Example of a pharmacological table containing only pointers to contents in the body of the patent documents. Columns in this table are organized by data type (i.e. different activity range) (*EP2049474B1 Table* 2)

Chemical patents typically present novel compounds, either specifying the chemical structure of compounds in the form of an image or through their systematic chemical name in the text, for which state of the art name-to-structure tools such as OPSIN [3] and MarvinSketch [4] can be used to reliably generate the structure. However, to back up the invention’s claims, patents also contain additional information related to these compounds—characterising them further, such as physical or spectroscopic data (Fig. 1a), information related to their preparation (Fig. 1b), or by exemplifying their claimed use through further information or numerical data. In addition to natural language text descriptions, such information is also presented in the form of tables and lists presenting the data in a compact and highly organized way. In fact, numerical data of very high interest to researchers, such as novel pharmacological results, are typically presented in this structured form [5].

In this context, manual excerption (or extraction) of key knowledge of compounds and their reactions from tables in chemical patents has been undertaken for large commercial chemical knowledge databases such as Elsevier’s Reaxys^®^ database^1^, providing reliable and comprehensive data within the domain of chemical literature and patents. The information provided by these commercial resources is of high-quality, but they are very expensive and time-consuming to build. As the number of new patent applications has been drastically increasing [6], it is infeasible for researchers and commercial chemical database providers to extract all of the relevant information manually from patent documents.

To reduce the time and effort needed for information extraction from chemical literature and patents, several rule-based text mining approaches have been developed. ChemDataExtractor [7] is an open-source toolkit for information extraction in chemical literature. The table parser module of ChemDataExtractor is able to identify components of tables (e.g. caption, header, footnote, data and units) with a tailored pre-processing pipeline and rule-based grammars. LeadMine [8] is a commercial tool that also supports information extraction from tables using rule-based methods. However, these tools have not been rigorously evaluated in terms of accuracy, efficiency and generalizablity on chemical patent data due to the lack of publicly available datasets like ChemTables.

Recent advances in machine learning-based Natural Language Processing techniques have also been adapted to the chemical domain, covering a wide range of information extraction tasks, including named entity recognition and relation extraction [9–14]. Most of these methods focus on processing plain text by leveraging state of the art Natural Language Processing (NLP) approaches, and tabular data is usually ignored or discarded. This leads to significant loss in the amount of compound-related information that can be extracted from patents. A key reason for ignoring tabular data is the lack of existing publicly available chemical patent corpora with gold standard annotations on tables.

In this paper, we therefore present a novel dataset—the ChemTables corpus—consisting of tables extracted from chemical patents augmented with gold standard annotations of semantic types reflecting the key content of each table. This dataset enables further research in information extraction from chemical tables. We make this dataset publicly available [15].

It is challenging to develop text mining approaches for the extraction of information from tabular data in chemical patents. Firstly, the number of tables and the average size of tables in chemical patents are much larger than in other contexts such as the web. For example, in the Web Data Commons Web Table Corpus 2012^2^ [16] which consists of tables crawled from the web, the average number of rows is 12.41, respectively, whereas in our ChemTables dataset, the average number of rows is 38.77, 3 times more than in web tables. Thus, although a wide range of methods and datasets for text mining in web tables have been proposed, the performance of these methods might be compromised when applied to tables in chemical patents.

Furthermore, as shown in Fig. 2, tables containing different types of information are often structured differently and not all tables contain valuable data relevant to key chemical compounds described in the patents. Therefore, it may be most effective to develop different approaches for extracting information from tables that are specific to the semantic type of the table. This means that table classification and identification of tables containing valuable data are fundamental steps to enabling high quality information extraction from tabular data within chemical patents.

In addition to introducing the ChemTables data set, we provide here an empirical comparison of several strong baseline approaches to table classification using this corpus, including conventional machine learning models based on the Naïve Bayes (NB) and Support Vector Machine (SVM) algorithms, as well as neural models TabNet [17], ResNet [18] and Table-BERT [19]. The experimental results show that all neural methods outperform the conventional machine learning baselines. Among the three neural models, Table-BERT produces the best classification performance which we ascribe to the power of pre-trained language models. We also examine potential issues specific to classifying chemical patent tables, and finally identify directions for further improvements in the classification performance.

## Related work

In this section, we summarise previous efforts to apply machine learning methods to tabular data, including table layout classification (section Layout classification of web tables), table question answering (section Layout classification of web tables) and table fact verification (section Table fact verification) and discuss how methods developed for these tasks can guide our approach to semantic classification task for chemical patent tables.

### Layout classification of web tables

Tables can be extracted from HTML format web pages by extracting content from elements surrounded by a $\texttt {<tables>}$ tag. These tables are structured with different layouts, such as vertical lists or matrices. A layout classification task can be defined, which aims to automatically identify the layout category of given web table (e.g. horizontal relational, vertical relational, matrix). We can determine the position of table headers and data cells more accurately if the layout of table is known. This task is fundamental for downstream table processing tasks. For example, in relation extraction, the column/row headers are often the most informative clue for identifying relations between two data cells in the same column/row. This task is challenging as web tables from diverse sources can have a huge vocabulary, making heuristic methods infeasible. Rule-based table classification methods based on selected keywords/tags often provide high recall but lack precision [20].

Here, we review several methods and datasets related to this task; these methods are directly relevant for our problem of classifying tables and several will be used as baseline methods in our experiments.

*TabNet* [17] is a supervised learning model for web table layout classification, and one of the earliest attempts to apply neural networks for image processing to the task of understanding table structure. It uses a Long Short Term Memory (LSTM) [21] network method to encode the sequence of tokens (words) within each table cell with embedding vectors. The encoded table is then treated as an image and fed into a Residual Network [18] to derive a latent representation of the table. A linear transformation followed by a soft-max function is applied on the latent representation for generating a probability distribution over all classes.

This model and the baselines it compares to are evaluated on a web table dataset, built by extracting tables from the top 500 web pages containing the highest numbers of tables in a subset of the April 2016 Common Crawl corpus [22].

Tables in this dataset are categorized based on the logical structure of the table, such as *Vertical Relational* and *Horizontal Relational*.

The experimental results show that TabNet outperforms baseline models which are based on Random Forest with handcrafted features [20, 23, 24], and on the other hand on the bidirectional HAN—Hierarchical Attention Network [25] neural model. This work also shows that an ensemble of 5 TabNets also outperforms an ensemble of 5 HANs and bidirectional HAN. This work shows that adapting models designed for image classification to tables in which cells are encoded as vectors of uniform size can outperform non-neural models with hand-crafted features engineered specifically for the table layout classification task.

*TabVec* Unsupervised methods have also been developed for table layout classification. TabVec [26] learns the semantic of tables using a vector space model based on random indexing [27]. Four different types of contexts are used for learning word vectors, including the text within each table cell, text in column/row headers, text in adjacent cells and the text surrounding the table. Based on the idea that each cell in the table represents the same concept, the cell vector is then calculated by taking the median of word vectors of all tokens in the content. Instead of proposing a specific label set for annotated tables, only a general concept of table types is defined. In this work, a table type is defined as the way how different concepts are organized in a table. Hence, a table type can be measured by calculating semantic consistency across rows, columns and the entire table. The semantic consistency is grounded by taking the deviation from mean and deviation from median at both row/column and table levels. The derived deviation vectors are then concatenated to form the final table vector representing the semantics of the table.

Since no label set is provided in this task, a K-Means clustering is applied on the vector representations of tables in the dataset. The label of each cluster is manually assigned by users, which eventually results in a label set similar to [17]. The proposed TabVec method is evaluated on 3 web datasets crawled from the domains of human trafficking advertisements, fire arms trading, and microcap stock market. In addition, a random sample of the July 2015 Common Crawl corpus is also used for comparison with other methods which focus only on generic domains. The experimental results show that the proposed TabVec method significantly outperforms TabNet on all 4 datasets, indicating that contextual information can be particularly helpful for learning table semantics. Table2Vec [28] uses a similar idea as TabVec, except that the embeddings are trained using the Word2Vec skip-gram model [29], and used for row/column population and a table retrieval task instead of classification.

To classify the content of tables in chemical patents, we focus on interpreting their content and semantics, instead of relying on purely structural information. Therefore, methods developed for (web) table layout classification need to be adapted to cater for this more semantically-informed classification task. They also need to be specifically evaluated over chemical patents.

### Table question answering

Question answering (QA) is the task of extracting answers to user questions from large document sets. It is a hard task and under active research in the NLP community as it requires a deep understanding both user questions and the documents where their answers may lie. Research in this area mainly focuses on answering questions based on unstructured text only. However, in many documents, the information necessary to answer user questions is described in tables. Thus, to build a well-rounded question answering system, the ability to extract answers from content within tables is needed. In the table question answering task, the goal is to answer a given question using data within a table.

*Compositional Semantic Parsing* [30] presented the WikiTableQuestion dataset, consisting of 22,033 natural question-answer pairs over 2108 tables extracted from Wikipedia pages. In this work, a novel method based on semantic parsing for table QA is proposed. The tables are first converted to knowledge graphs in which table cells are entity nodes; table rows are row nodes; table columns are directed edges from the row nodes to entity nodes of that column. Based on the knowledge graph converted from table, the questions are parsed to a set of logical forms. The logical forms of questions can be executed on the table knowledge graph as queries to retrieve the answer.

The authors proposed a novel semantic parsing method *Floating Parser* to address the difficulty of table-based parsing, such as the mismatch between words and utterance/predicates. This parser uses a relaxed lexical rule for anchoring predicates in logical forms to tokens by replacing the fixed span in chart parsing by a floating cell which only restricts the category and size of logical form.

The logical forms derived from tables and questions are fed into a log-linear model which optimizes the probability of the selected logical form retrieving the correct answer after execution on the table knowledge graph. Features used in this model include word *n*-grams in both question and table, the headword of the question (e.g. what, who, how many, etc) and the type of the answers (e.g. NUM, DATE, ENTITY) retrieved by using the logical form-table pair.

Haug et al. [31] proposed a CNN-based neural method for table QA task. In this approach, logical forms are first extracted using the same method as [30]. Then the logical forms are naturalized to a plain textual representation by applying a novel algorithm which recursively traverses the $\lambda -DCS$ logical form derived from the previous step.

The GloVe vectors of tokens in the naturalized logical forms are feed into 2 jointly trained CNNs for obtaining sentence-level embeddings. The final answers are then selected based on the neural similarity between the sentence embeddings of the logical form and the question. This work also evaluated several ways to calculate the similarity of sentence embeddings on this task, including dot-product, Bi-Linear (BILIN), and fully connected Feed-Forward networks (FC) applied on the concatenation of 2-sentence embeddings. The experimental results show that using the weighted average of BILIN and FC similarity and an ensemble of 15 models results in a performance exceeding [30] 1.6% in absolute accuracy. This work shows that neural models using semantic information from pre-trained embeddings with natural language input can produce better performance than a model based on logical forms and hand-crafted features.

Krishnamurthy et al [32] proposed a neural encoder-decoder architecture for semantic parsing in tables. In addition to the word embeddings of each token in the question, an entity linking embedding is also introduced in this model. To construct the entity linking embeddings, similarity between pairs of entity mentions in the knowledge graph and tokens in question is measured. The entity linking score consists of two parts, the similarity between word vectors of entity and token in questions and the output of a linear classifier built on hand-crafted features such as exact/lemma match, edit distance and NER tags. The entity linking score across all entities of the same type is then fed into a softmax function. Based on the resulting probability distributions, the weighted sum of embedding vectors of all entities forms the final entity linking embedding. To avoid the problem of ignored type constraints imposed by previous neural semantic parsers, strong type constraints are applied by only allowing the decoder to generate results in a grammar that guarantees well-formed logical forms.

The experimental results show a significant improvement based on previous state-of-the-art results [30, 31]. An absolute improvement of 7.2% in accuracy is observed comparing the CNN-based approach proposed by [31], showing that generating logical forms using a neural parser can produce logical forms more accurately than chart parser optimized for semantic parsing in tables.

In summary, most table QA methods take the same strategy as knowledge base QA in which tables play the role of a knowledge base. In these methods, tables and questions are first converted into logical forms by semantic parsing, and then the answer is extracted by querying the table. Since pre-defined logical forms and rules are required to perform table semantic parsing, such constraints might cause unexpected loss of information during the conversion process. In table classification, our objective is to obtain an accurate semantic representation of the table. Since there is no need for pairing questions with table contents, semantic parsing may not be the ideal way to extract semantic information from tabular data as there is some loss of information.

### Table fact verification

Similar to table QA, table fact verification also takes as input a table and statement pair. However, instead of extracting answers from the table, table text entailment models seek to determine whether a statement is true based on the data presented in a table.

*Table-BERT* [19] captures contextualized table semantics by applying BERT [33] on the concatenation of linearized tables and the statement to be verified. In this work, two different approaches to linearization are proposed. The first approach, serialization, simply concatenates content of all table cells with *[SEP]* (a special token which separates different sentences in BERT’s input). Under this setting, the position count of each token is reset to 0 at the beginning of each cell. The second approach, naturalization, uses a template filling method in which the content of each cell is enriched by adding the column and row headers.

The concatenation of linearized tables and the statement is then fed into a pre-trained BERT for extracting semantic representations. The same method for extracting sentence-level semantic representation in BERT is used for this task. The derived representation of tables will be fed into a linear layer with a binary classification objective to make a prediction on whether the table supports the given statement.

The experimental results show that Table-BERT performs significantly better than strong baselines, including a weakly-supervised latent program analysis approach (similar to [34]), showing that semantic information captured by pre-trained language models can improve upon semantic parsing. Moreover, pre-trained language model-based approaches also require less effort in adapting semantic parsers designed for other tasks to tabular data. Among different settings of Table-BERT, the naturalization approach outperforms the serialization approach by a large margin since more context can be incorporated for each table cell.

Table-BERT is capable of encoding the combination of tabular data and the statement into vectors that can be used as features for classification tasks, which means that Table-BERT can be used as a semantic encoder for tables, and especially if we use flattened tabular data as input. In the table verification task, the semantic representations of both table and the statement need to be obtained to identify whether the table entails the statement. Since our table classification task also requires semantic representations of tables, it is worth investigating whether Table-BERT can be adapted to table semantic classification task using only tables as input.

### Bridging quantities between tables and texts

The goal of this task is to identify co-references between quantities mentioned in tables and in the main body of documents. Besides direct mentions (i.e. values in a table are identical to those mentioned in body text), aggregations of values also need to be identified in this task. Hence, the first step of this task is to extract all pairs of quantity mentions in tables and texts. Then pairs which co-refer can be identified through a binary classification task. This task requires modeling of table content within cells as well as consideration of the broader document content outside of the tables.

*ExQuisiTe* [35, 36] proposed a multi-stage system for linking numeric mentions in tables and texts. Firstly, all quantity mentions are extracted from both and tables and texts. In this stage, aggregation of values such as total, difference, percentage and change ratio between values in the same column/row are also calculated and add to the collection of quantities.

In the second stage, all possible pairs between quantities are enumerated and fed into a Random Forest-based classifier to eliminate the pairs that are not likely to be relational. Features used for building this classifier include context features, such as word and phrase overlaps and quantity features such as relative difference between values, unit match and aggregation function match. After pruning, the remaining candidate mention-pairs are classified based on whether the text mention is an aggregation value or a single-cell mention using indicator words, exact matches in tables and other context features.

Heuristic approaches are also considered in this work. Value and unit mismatches are discarded after ensuring mention type matches. Finally, the top-*k* candidate pairs will be selected for the next stage of processing. In this system, the selection of value *k* depends on the distribution entropy of the confidence score returned by the classifier for pairs with the same text mention.

CCTR-83 [37] is a corpus specifically built for information extraction tasks in tables. It consists of 83 tables in 43 biomedical documents. Tables in this dataset can be divided into 2 groups of roughly equal sizes by their topics which are human cancer and mouse genetics (43 and 40 respectively). When constructing the dataset, similar cells are first grouped into *cell groups* and the terms which correspond to concepts in UMLS-NCI schemes will be annotated. The type of cell groups are decided based on the common parents of concepts presented in this group. Finally, suggested relations between cell groups will be generated based on matches to UMLS Metathesaurus and UMLS SN, which the annotator will decide whether to accept it as an annotation or not. In this dataset, tables are annotated by experts with post-graduate degree in Biology. Thus, this dataset has a very high inner-annotator agreement of Kappa values of 0.88, 0.87, 0.82 for concept, cell type and relation annotations, respectively. However, owing to budgetary constraints, it is small in size and may not be sufficient for complex machine learning methods. In this dataset, it is possible to do classification based on semantic types by using topic of tables (human cancer and mouse genetics) as labels. However, the number of table instances is too small for any supervised machine learning approach.

## ChemTables Corpus

In this section, we present the annotation guidelines and data preparation process of our ChemTables dataset (section Corpus definition), general statistics of the dataset (section Annotation statistics), and more detailed dimension-level (section Dimension-level statistics) and cell-level (Fig. 6) statistics. Finally, we present the standard data split we use for evaluating our table semantic classification methods (section  Data split for evaluation).

### Corpus definition

To enable automatic determination of semantic type and relevancy of tables in chemical patents, we have constructed a new table dataset named ChemTables. In contrast to WikiTableQuestions and other datasets that are built from web crawls, our ChemTables corpus focuses exclusively on tables in chemical patents, and makes use of a categorization scheme directly relevant to chemistry.

For the purpose of identifying tables containing data valuable to research, the taxonomy of tables must be well defined so that the relevancy of tables can be assessed based on their label. In order to reflect the categories of content that are of interest for researchers, we adapted the categorization system for facts in patents from Reaxys^®^ [38], a highly curated database of experimental facts derived from relevant literature including patents. The storage of data follows strict organising principles. The same organisation is used in the manual excerption process. The main purpose of patent excerption for Reaxys is to identify and capture significant data about compounds and their related reactions and facts in a reliable and comprehensive way. Reaxys has had positive reception from chemists as it can help accelerating the search of property information of chemical compounds and provides the ability to compare data from different sources [39, 40].

Hence, we assume that the key data types derived from Reaxys for the ChemTables labelling guidelines represent the most typical and important types of data in chemical patents.

**Table 1:** Table categories IDs within the Reaxys scheme and examples of expected content

Label	Description	Examples
SPECT	Spectroscopic data	Mass spectrometry, IR/NMR spectroscopy
PHYS	Physical data	Melting point, quantum chemical calculations
IDE	Identification of compounds	Chemical names, structure, formula, label
RX	All properties of reactions	Starting materials, products, yields
PHARM	Pharmacological data	Pharmacological usage of chemicals
CHEM	Chemical data	Catalysis, electrochemical reactions
COMPOSITION	Compositions of mixtures	Compositions made up by multiple ingredients
PROPERTY	Properties of chemicals	The time of resistance of a photoresis
OTHER	Other tables	–

**Table 2:** Inner annotator agreement between annotator group 1, 2 and gold set in precision (${\mathcal {P}}$), recall (${\mathcal {R}}$) and Macro $F_1$ score (${\mathcal {F}}_1$)

Label	Annotator 1			Annotator 2			Random		
	${\mathcal {P}}$	${\mathcal {R}}$	${\mathcal {F}}_1$	${\mathcal {P}}$	${\mathcal {R}}$	${\mathcal {F}}_1$	${\mathcal {P}}$	${\mathcal {R}}$	${\mathcal {F}}_1$
SPEC	97.20	97.74	97.47	96.77	96.67	97.47	24.27	26.14	25.17
PHYS	79.02	89.13	83.77	88.43	89.15	88.79	9.43	3.79	5.41
IDE	88.47	94.51	91.39	94.04	85.54	89.59	16.64	18.87	17.68
RX	62.32	82.45	70.98	80.68	83.72	82.17	7.14	4.84	5.77
PHARM	80.94	93.67	86.84	87.55	91.69	89.57	14.76	17.42	15.98
COMPOSITION	85.71	85.41	85.56	79.20	79.94	79.57	6.97	8.04	7.47
PROPERTY	45.56	61.89	52.49	36.68	46.35	40.95	3.13	3.45	3.28
OTHER	69.92	25.05	36.88	62.21	58.37	60.23	13.43	15.32	14.31
Overall	76.14	78.73	75.67	78.20	78.93	78.54	11.97	12.23	11.88

“Random” refers to randomly sampled label from the label distribution in the final gold standard dataset

Following the Reaxys Excerption Guideline, in our ChemTables dataset, tables in chemical patents are categorized based on their semantic types as listed in Table 1. We identified the 5 most relevant types of information for chemical research (rows 1 to 5 in Table 1). Since there can be different types of data presented in the same table, a single table can be assigned to multiple categories within the annotation scheme. However, if a group of data in the table cannot be categorized into any class in the Reaxys schema, only one of three out-of-schema category labels (COMPOSITION, PROPERTY, OTHER) will be assigned following the order of priority shown in the table.

### Corpus construction

We first sampled 1K XML-formatted patents from the European Patent Office (EPO) and the United States Patent and Trademark Office (USPTO). The original files were derived from the repositories of a 3rd party database but the same can be obtained from USPTO/EPO bulk download service. These patents must have at least 1 table to be retained. IPC classes were used for filtering; we selected patents from the classes C07 (Organic Chemistry), C09B (Dyes), A01N (Agrochemistry) and A61K (Drugs). If a patent has been published more than one time in different patent offices (known as a *patent family* of related patents), we only use the earliest instance of this patent within the patent family. Tables are automatically extracted from these patents using a toolkit developed internally. However, since the patents from EPO and USPTO in their XML format are available for download, this process can be replicated by extracting textual content from XML tags corresponding to tables (i.e. $\texttt {<tables>}$) using any publicly available XML parser. We have made a comparable tool available^3^.

On average, there are more than 8 tables per patent. To achieve better downstream task performance, after extraction, we tokenize the textual content in each cell with a chemical tokenizer which is a component of the chemical named entity recognition (NER) tool OSCAR4^4^. [41]. This process results in a total of 788 tables consisting of 3 million tokens^5^. In the ChemTables dataset, we store all tables extracted from a patent document in different worksheets within a single Excel (.xlsx) file. The original patents in both XML format and PDF format are provided with the table file, which means that tables in this corpus can be linked back to its context in the original patents by matching the caption. The 788 annotated tables are randomly sampled from the data extracted.

This table dataset was sent to 3 groups of Reaxys excerptors who hold at least a Master’s degree in chemistry. The excerptors have years of experience working on Reaxys excerption, thus are very familiar with relevant topics and concepts in the chemical patent table annotation task. Two annotator groups (Annotator 1 and 2 in Table 2) annotated the ChemTables dataset independently while the third annotator group worked to merge annotations from different annotators and make final decisions in case of disagreements. We then use the harmonized set as the final gold standard dataset. We use $F_1$ rather than Kappa score to measure agreement, as the distribution of labels is highly skewed [42]. The inner-annotator agreement (IAA) scores in terms of $F_1$ score are computed by comparing the annotations of Annotators 1 & 2 against the gold set.

As shown in Table 2, both annotator groups achieve a high $> 80\% F_1$ score on average. This confirms that the annotation of ChemTables dataset is of high quality. Among all labels, SPEC, PHYS and IDE gets 80+ $F_1$ score in both annotator groups. Since spectroscopic and physical data are of greater interest to chemical researchers, it is not surprising that the annotation of these 3 labels has a higher level of consensus than the others. Among labels with comparatively lower IAA scores, OTHER and PROPERTY stand out as much lower than other categories. These labels are only used when the table is considered “Out of Reaxys Scheme”. This makes it difficult to disambiguate these tables against tables which are within the “Reaxys Scheme” but may contain similar information.

**Fig. 3:**
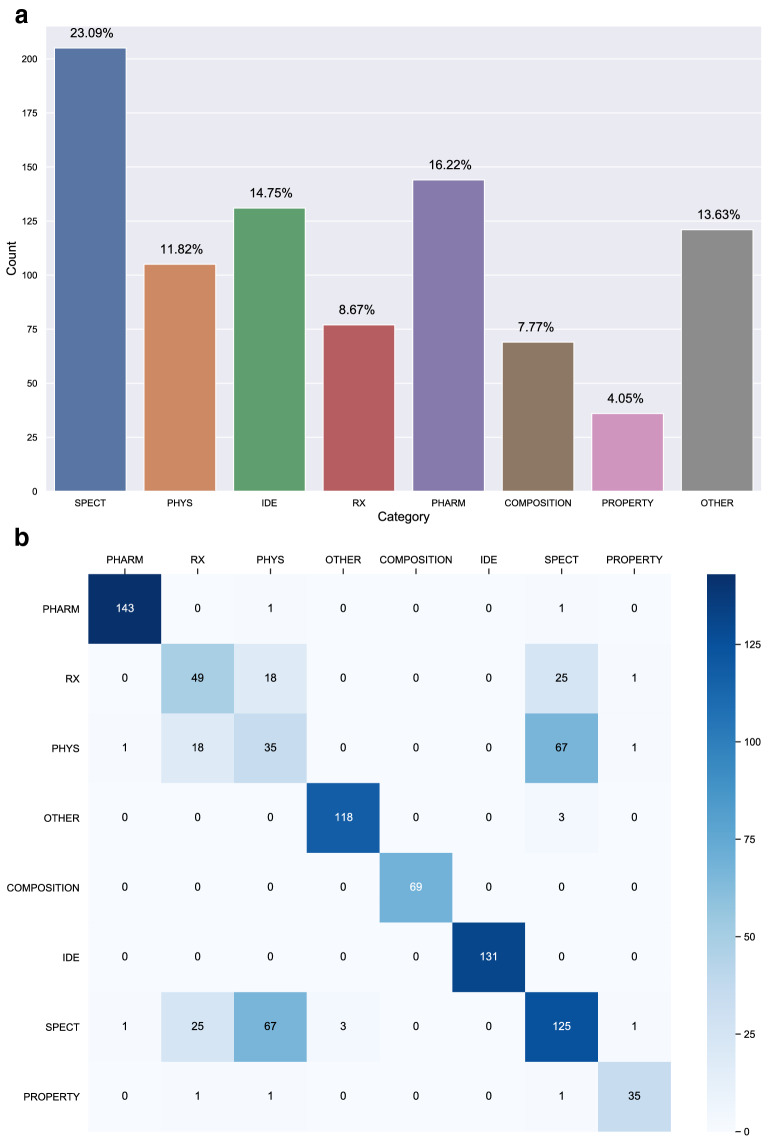
Statistics on annotations per semantic category. **a** Statistics on total number of annotations per semantic category. **b** Statistics on category label overlaps

**Fig. 4:**
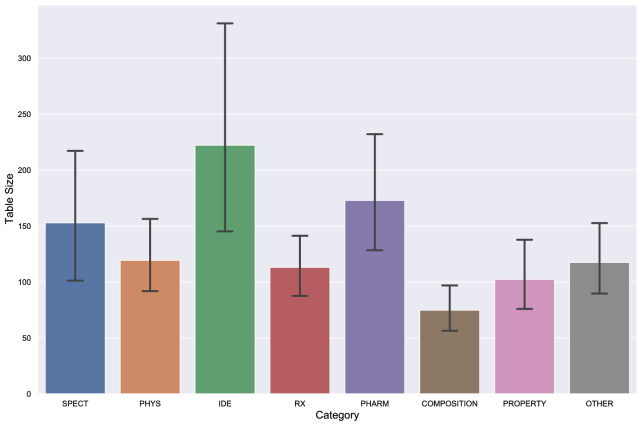
Statistics on table size per semantic category. Table size is measured as the product of number of rows and columns. The whiskers show a 95% confidence interval

### Annotation statistics

In this section, we show statistics over annotations in the dataset. Figure 3a shows the count of annotations per semantic type. Among 8846 annotations, SPECT is the most common label as 23.1% of all tables in ChemTables contain spectroscopic data, while only 4% (37 instances) tables are annotated as PROPERTY.

As our annotation guidelines allows one table to be annotated with different labels, we also show the statistics of co-occurrences between labels. As shown in Fig. 3b, most overlaps occur between RX, PHYS and SPECT, indicating that physical, spectroscopic and reaction related data are usually presented in the same table.

### Dimension-level statistics

In this section we show statistics related to table size. Merged cells which take up the space of multiple data cells are frequently used in headers that summarise the semantic of multiple columns/rows. During the extraction process, information about merged cells is not preserved. Thus, only one of the original cells which forms the merged cell is used to store the content of the merged cell and other cells are left empty. In addition, rows which are shorter than the longest row in the table are padded with empty cells to ensure all rows in the same table have the same length. We measure the size of tables by taking the product of number of rows and number of columns. Figure 4 shows average size of tables per semantic category

**Fig. 5:**
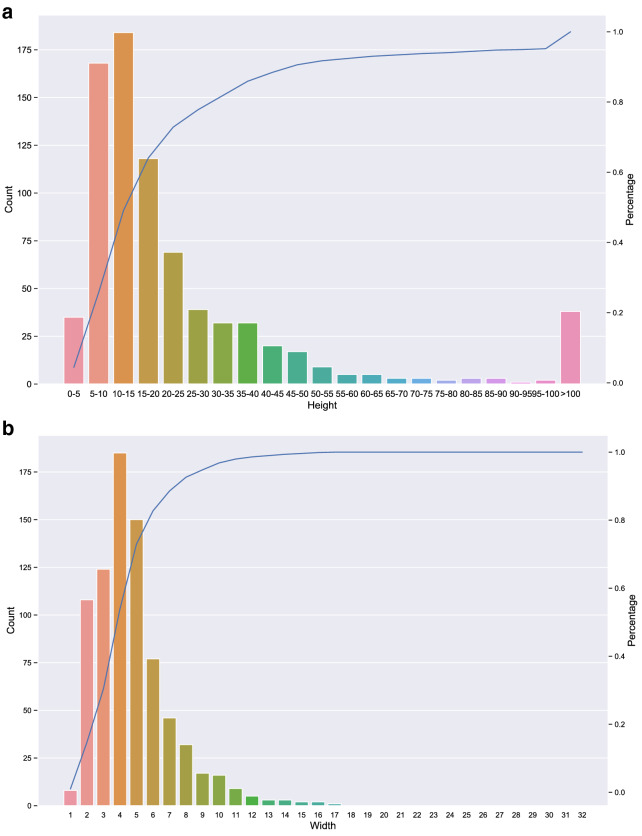
Statistics on size of tables in the dataset (*y*-axis on the right side shows percentage of instances with number of rows/columns less than certain range.) **a** Statistics on number of rows. **b** Statistics on number of columns

**Fig. 6:**
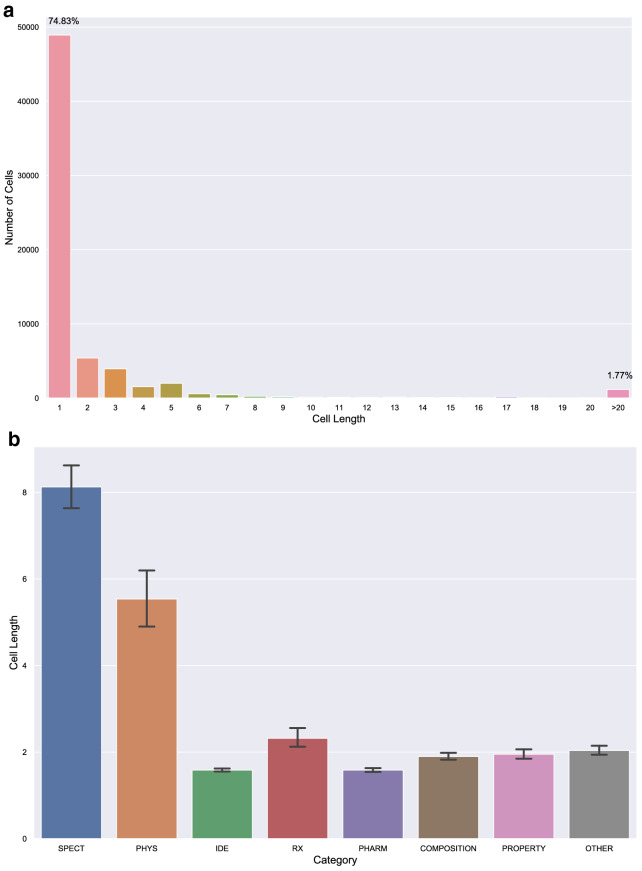
Statistics on length of cells within tables in the dataset. **a** Statistics on number of tokens in cells. **b** Average number of tokens in cells per table category (estimator shows 95% confidence interval)

We also perform fine-grained table statistics on row and column levels. As Fig. 5a shows, the range of table heights in the dataset is relatively wide, from less than 5 rows to more than 1000 rows. This is not surprising since patents usually contain many details, as the authors tend to maximize the scope of claims of their inventions. However, as Fig. 5a shows, only a small fraction of tables in the dataset (approx. 10%) have more than 100 rows, the height of approximately 80% of the tables in the dataset is smaller than 35.

The width of tables in patents shows completely different patterns than their heights. The range of numbers of columns is very narrow compared to that of rows, with the width of 99% of tables falling in the range from 1 to 15, while the maximum number of columns is 32. This implies that most tables in patents grow vertically (i.e. column headers control the content type of each column while rows represent different instances).

### Cell-level statistics

In this section we provide cell level statistics focusing on the length of text (number of tokens) in table cells. Statistic results in Figure 6 shows that most of the cells in tables are generally short as 74+% of cells contain only 1 token (usually data in a single number/word) and 98+% of cells contain fewer than 20 tokens.

Cells in tables with SPECT label have the longest average length (approx. 8 tokens per cell) while tables labeled with PHARM have the shortest average length of cells ($< 2$ tokens per cell).

Table captions usually contain some text summarising the content of the table. Therefore, the caption of every table in ChemTables dataset is also included at the first cell of the first row. If a table has no caption, we use “Unlabelled table *n*” as caption, where *n* is a numerical identifier. Thus, we also present cell-level statistics over captions, as they tend to have different properties compared to other cells. Indeed, the captions of 90% of tables in the dataset contain 2-4 tokens, indicating that table captions from chemical patents are much shorter and contain less information than table captions in scientific literature. These captions are usually only table identifiers, such as “Table 2” or “Unlabelled Table 1”. We also find that the average title length is very similar across all table categories. Noticeably, tables with label PHARM have the shortest average cell length but the longest average caption length, implying that information in pharmacological tables is usually more complex and thus needs longer texts to be conveyed.

### Data split for evaluation

As shown in Fig. 3b, the number of instances with multiple labels is low (10%). Among all tables with more than one type of category annotations, 82% instances are a combination of SPECT and PHYS. Hence, when pre-processing the dataset, we merge tables with both SPECT and PHYS labels into a new category SPECT|PHYS. For the remaining multi-label instances, we convert those into single-label instances by choosing the most frequent label.

We make the entire dataset publicly available to motivate further study on this task^6^ [15].

## Experimental methodology

We present our empirical study of table semantic classification over our ChemTables dataset. We first outline the non-neural baseline methods (section Baseline models), and then introduce the neural methods (section Neural network-based models), starting from networks taking 2-dimensional tabular data as input (section Cell embedder, TabNet and  TBResNet) and pre-trained language models which take flattened tables as input (section Table-BERT). We also detail our evaluation metrics in section Evaluation metrics.

### Baseline models

In this work, we compare state-of-the-art neural methods on table classification with two non-neural baseline models Naïve Bayes (NB) and Support Vector Machine (SVM), which use bag-of-words features. For each table, texts in its cells are tokenized using the OSCAR4 [41] tokenizer. We then calculate the value of each bag-of-words feature by using the TF-IDF weighting scheme. Similar to neural models, we also explore the effect of input size for baseline models. We use the results with optimal input size to compare with other models.

### Neural network-based models

Similar to images, tables are naturally 2-dimensional structured data, with table cells acting as counterparts to pixels in images. It thus seems possible to attempt generalizing well-developed methods in computer vision to the context of table processing. However, there are some challenges for applying computer vision methods to tables. One major obstacle is the difference between pixels and table cells. Pixels, in conventional image datasets, are represented in RGB encoding. Hence the size of the vectors representing pixels is the same. By contrast, text in table cells contain varying numbers of tokens. Thus, an embedder is needed to encode the textual content in table cells into vectors having a uniform number of dimensions. After embedding the table into a feature map, an image classification model can then be used as a decoder to determine the semantic type of a given table. In this work, we evaluate the residual network based neural model TabNet [17] that has been applied to the web table layout classification task, a more complex variation of a residual network [18], and a BERT-based model [19].

In an image dataset, the size of different images is usually the same, whereas tables are often vary substantially in size. Hence, to be able to apply image classification models on tables, we need to pad or truncate all tables in the dataset to the same size. We will explore the effect of input size on table classification performance in section Effect of input size. Since the size of more than $80\%$ tables in the dataset are within 32 by 32 as shown in Fig. 5, we only explore input size within this range.

**Fig. 7:**
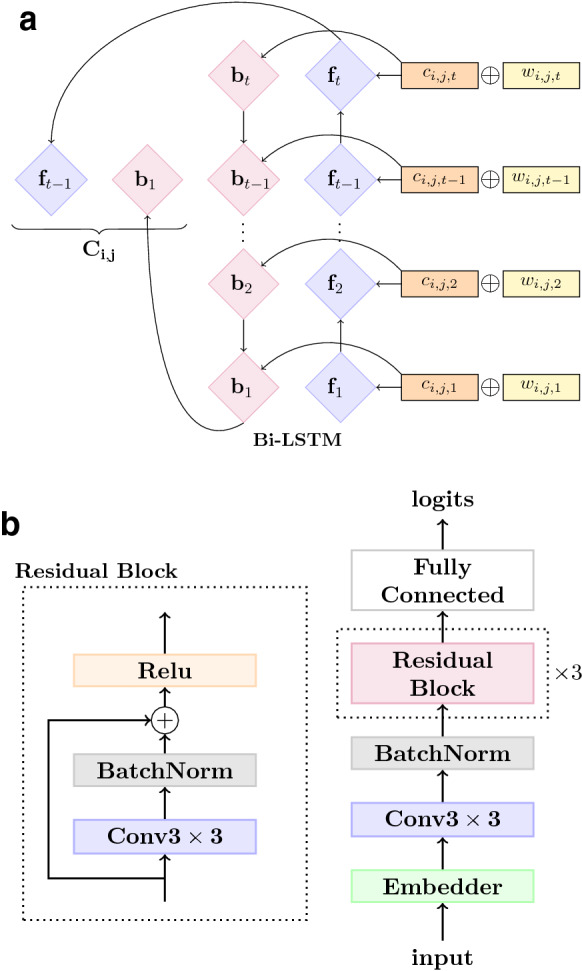
Model Architecture of TabNet. **a** Embedder architecture. **b** TabNet architecture

**Fig. 8:**
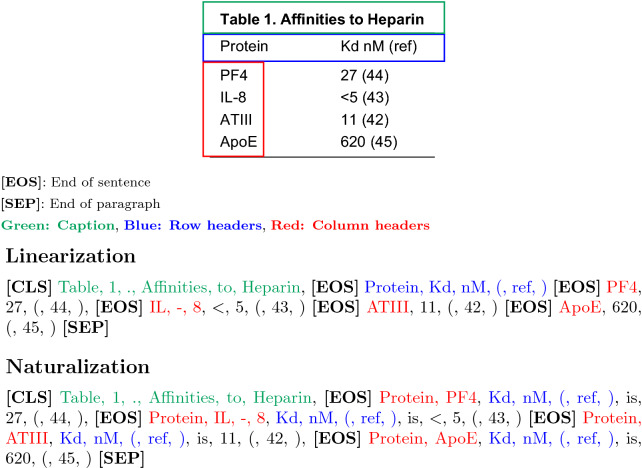
Illustration of different pre-processing approach used in Table-BERT

**Fig. 9:**
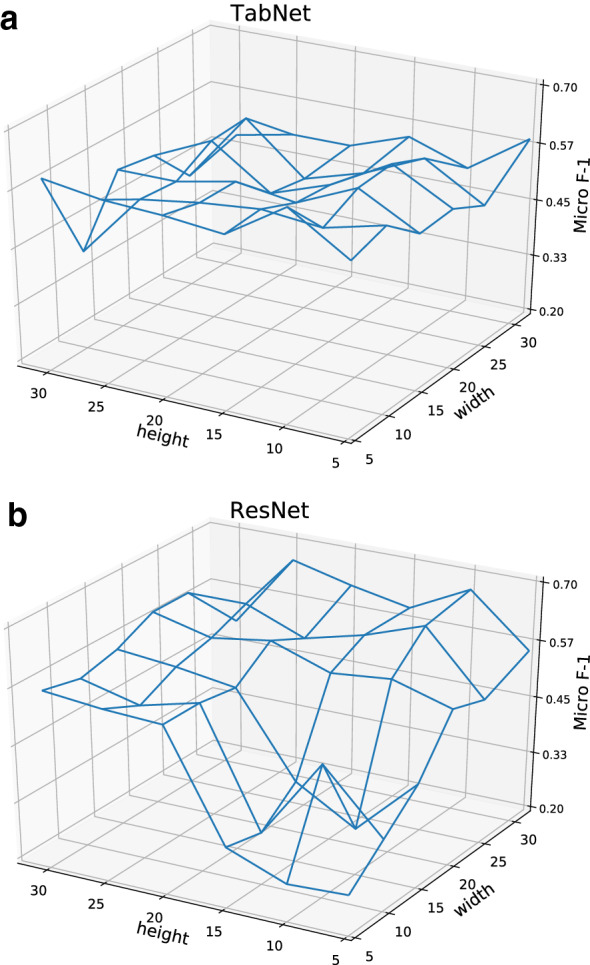
Effect of input length on classification performance of TabNet and TBResNet. **a** TabNet. **b** TBResNet

#### Cell embedder

We employ a Bi-LSTM encoder with a combination of word *w* and character-level *c* embeddings as shown in Fig. 7a. ChemPatent word embeddings are derived from a skip-gram model pre-trained on a chemical patent corpus [13]. We use fixed weights for tokens presented in the vocabulary of the ChemPatent word embeddings while using trainable weights for all out-of-vocabulary words that appear more than 5 times in the entire table training set. For character-level word embeddings, we employ CNN-based character-level word representations [43] with a kernel size of 3.

#### TabNet

In TabNet [17] a Bi-LSTM encoder is applied to the word embeddings of each token in table cells (w/o character-level word representation). Then, instead of concatenating the hidden states from both directions of its Bi-LSTM encoder, TabNet adopts the word-level additive attention used in [25] to calculate attention weights $a_{i,j,t}$ for the hidden states ${\mathbf {h}}_{i,j,t}$ of token *t* in the table cell located at the *i*-th row and *j*-th column, and then takes their weighted average sum as final cell-level representation.

Attention is then formulated as

1 $$\begin{aligned} \begin{aligned} \mathbf{u }_{i,j,t}&= \texttt {tanh}(\mathbf{W }_w\mathbf{h }_{i,j,t} + \mathbf{b }_w) \\ a_{i,j,t}&= \frac{\texttt {exp}(\mathbf{u }_{i,j,t}^{\top }\mathbf{u }_{w})}{\sum _t{\texttt {exp}(\mathbf{u }_{i,j,t}^{\top }\mathbf{u }_{w})}} \\ \mathbf{c }_{i,j}&= \sum _t{a_{i,j,t}\mathbf{h }_{i,j,t}}, \end{aligned} \end{aligned}$$

where ${\mathbf {u}}_w$ is a trainable context vector and ${\mathbf {W}}_w$ is a projection matrix which maps the hidden states to the same dimensionality as the context vector.

The cell-level representation ${\mathbf {c}}_{i,j}$ is then fed as input to a $3\times 3$ convolutional layer which is followed by 3 consecutive residual blocks. The residual blocks are illustrated in Fig. 7b, where $x_l$ refers to the output of the *l*-th layer of a residual block.

#### TBResNet

In TabNet, although residual blocks are used for encoding tables, the size and depth of the model is still not comparable with state-of-the-art deep residual networks for image classification. Hence, to investigate the effect of increasing model complexity on table classification performance, we use a 18-layer ResNet [18] for table classification. TBResNet takes the feature map generated by the cell-level embedder and feeds it as input to a convolutional layer, which down-samples the output to match the input dimension of ResNet18 [18].

#### Table-BERT

Table-BERT [19] was proposed for the table fact verification task. The goal of this task is to verify whether a table entails a textual statement which describes facts in the table. In contrast to TabNet and TBResNet, BERT is a language model pretrained on massive un-annotated plain text corpora. Hence, tables must be flattened into sequences of sentences before being used as input to BERT. Table-BERT then takes a pre-trained BERT model as starting point and uses the concatenation of the flattened table and the statement as input to fine-tune BERT on a binary classification objective. There are two different methods for flattening tables proposed and evaluated in this work [19], namely linearization and naturalization.

*Linearization* The linearization approach simply takes the concatenation of tokens within all cells in the table to form a paragraph which will be used directly as the input to BERT. The cells are added to the paragraph following a top to bottom and left to right order. Each row here is regarded as sentence and will be separated by a ‘.’ which represents the end of sentence. There is no separation added between content from neighboring cells in the same row.

*Naturalization* is a template filling approach. In this approach, row and column headers of each row are incorporated into each cell, which make the sentence structure of the flattened text more natural and provide extra semantic information about the data within each table cell. Compared to tables from Wikipedia where the determination of column and row header is relatively trivial, the heterogeneity of chemical patent tables makes it difficult to accurately locate the headers (see section Effect of flattening strategy for detail). Here we assume that the first non-repeating row in a table is the column headers (here, captions may be repeated in the first row of patent tables), and the first column as row headers. In this approach, the column headers and row headers are incorporated within each cell (e.g. “The name of row *[row_id]* is *[row_header]* and its *[column_header]* is *[cell_value]*.”) instead of being added to the paragraph individually.

Figure 8 shows examples of the two approaches for flattening tables. Comparing to the WikiTableQuestion corpus on which Table-BERT was first evaluated [19], tables in the ChemTables dataset do not have explicit annotations of the location of header row/columns. Therefore, we take the first non-empty row under the table caption as row headers and the first column except the table caption as column headers. Then, we fine-tune BERT on for the table-classification task.

The table fact verification task is a binary classification task and its input a statement-table pair. Hence, to adapt this model to our table semantic classification task, we use the flattened table only as input and change the size of the output layer from 1 to the number of labels in the ChemTable dataset. Since flattened tables are usually longer than the input size limit of BERT which is 512 sub-tokens, we explore the effect of limiting the length of a flattened table on classification performance, the results of which are presented in section Effect of input size.

### Evaluation metrics

We use a stratified 60:20:20 split for training, development and test set. We use micro-averaged $F_1$ score across all classes over development set as indicator for performance improvement. All models in this paper are trained for at most 50 epochs, and early stopping is applied if there are no micro-averaged $F_1$ score improvements observed after 5 epochs. Macro and weighted average $F_1$ scores across all labels are also reported.

## Results

In this section, we present our main experimental results comparing different neural methods on table semantic classification with our baselines (section Main Rresults). We then discuss how change in input size impacts the performance of neural models (section Effect of input size), and the effect of different pre-processing strategies (section Effect of flattening strategy). We show that the tables in the ChemTables dataset are sufficient to train state-of-the-art machine learning methods. We also provide analysis on error cases and propose possible ways to further improve classification performance (section Error analysis).

**Table 3:** Table classification baseline results in $F_1$ measure. “Count” denoted number of instances in the entire ChemTables dataset

Category	NB	SVM	TabNet	TBResNet	Table-BERT	Count
SPECT	81.36	82.76	85.71	92.59	**96**.**30**	138
PHYS	**66**.**67**	42.11	0.00	0.00	23.53	38
SPECT|PHYS	64.00	69.23	78.57	**88**.**00**	84.62	67
IDE	75.47	69.23	84.00	77.42	**96**.**15**	137
RX	**73**.**68**	43.48	35.29	28.57	**73**.**68**	49
PHARM	76.67	69.23	68.75	66.67	**82**.**76**	143
COMPOSITION	**75**.**86**	60.61	62.50	74.07	74.07	69
PROPERTY	**54**.**55**	37.50	25.00	0.00	30.00	35
OTHER	54.90	45.71	46.81	40.82	**58**.**54**	118
Micro Avg.	60.54	59.72	65.61	66.24	**76**.**43**	–

**Table 4:** Micro Avg. $F_1$ scores of models with only table caption and row headers as inputs. “# of Rows” denotes the number of rows included as input for the models ($1 =$ only the header row used as input)

Metric	Micro $F_1$			Macro $F_1$			Weighted Avg. $F_1$		
# of Rows	1	2	3	1	2	3	1	2	3
NB	19.24	29.40	37.21	18.08	27.88	36.56	22.33	35.12	40.73
SVM	**27**.**20**	**35**.**72**	**38**.**88**	**22**.**17**	**33**.**10**	**37**.**18**	**23**.**47**	**38**.**78**	**41**.**65**
TabNet	**40**.**12**	**49**.**04**	**50**.**95**	**32**.**09**	**43**.**30**	**42**.**26**	**37**.**67**	**46**.**99**	**49**.**19**
ResNet	37.58	42.68	44.59	26.78	32.39	34.35	33.38	40.34	42.39
Table-BERT	34.39	40.76	48.41	25.51	34.51	42.23	30.97	38.67	46.93

### Main results

Table 3 shows table classification performances for baseline models (Naive Bayes, SVM) and neural network models (TabNet, TBResNet and Table-BERT). The first 9 rows show the micro-averaged $F_1$ score per each semantic type while the last row shows the overall performances in micro-average $F_1$ scores.

For baseline methods, the SVM and Naive Bayes have very similar overall performance. SVM outperforms Naive Bayes on categories with more than 100 samples except for IDE, whereas Naive Bayes archived highest score among all models on under represented categories (i.e. PHYS, RX, COMPOSITION and PROPERTY). Comparing the baseline approaches with the neural based methods, all neural based methods outperform SVM by at least 5 points in the micro-average $F_1$ score. Among the 3 neural models, Table-BERT achieves the best overall performance, with TBResNet second.

Regarding performance broken by semantic type, labels with more than 50 instances in the entire dataset achieve greater than 70 micro-averaged $F_1$ score, except the label OTHER. For the least frequent labels PHYS and PROPERTY, Naive Bayes obtains micro $F_1$ scores much higher than those of other models. This suggests that a simpler model is more advantageous in such extreme low resource condition.

The label OTHER shows a different pattern compared to the remaining labels. With 118 instances in the entire dataset, the best micro-average $F_1$ performance on this label is 54.90, achieved by Naive Bayes. This category is particularly hard to identify as it can contain any type of information, that may not moreover be relevant to the core invention being protected by the patent.

When comparing the performance by table class in Table 3, we found that advantages in performance for non-neural models mainly come from under-represented classes which have less than 50 instances in the entire dataset. Whereas neural models out-perform baseline models when there is sufficient training data, achieving overall a better micro-average performance.

### Effect of input size

As described in the dimension-level dataset statistics in “Dimension-level Statistics”, tables in our ChemTables dataset are of various sizes and the range of the table size is wide. To adapt neural models from image classification to this task, table instances within the same mini-batch need to be padded to the same size. For pre-trained language models such as Table-BERT, a limitation on input sequence length also applies. Hence, it is important to determine the input size yielding the best overall classification performance.

Table 4 shows the test performance of all models when we use no more than the first 3 rows as input (all columns are used in this set of experiments), to explore whether the semantic type of tables can be determined by caption and row headers alone. The experimental results show that with only the first 3 rows, classification performance drops at least 15 $F_1$ score points in all 3 metrics compared to the models using an optimal input size. Hence, although semantic information in captions and headers are denser, including the body of the table can still help to significantly improve the understanding of table semantics.

#### TabNet and TBResNet

Figure 9 shows classification performance in micro-averaged $F_1$ score under different combinations of input height (number of rows) and width (number of columns), both ranging within the set [5, 10, 15, 20, 25, 32] of values. Figure 9a shows that for TabNet the best performance is achieved when using 10 rows and 5 columns, while as shown in Fig. 9b for TBResNet, the optimal performance is achieved when using 10 rows and 32 columns. For models taking 2-dimensional table data as inputs, the classification performance does benefit from increasing model complexity as TBResNet outperforms TabNet in all 3 metrics in overall performance. From observing Fig. 9a, b, one can see that TBResNet is indeed able to learn more knowledge of table semantics from larger input sizes, which results in higher classification accuracy.

As shown in Fig. 9a, b, for TBResNet, the difference between the highest and lowest $\hbox {F}_1$ score is larger than that of TabNet. We can also observe that the performance of model drops faster than TabNet when input size deviates from the optimal value. This shows that as the model complexity increases, the performance of ths model is more sensitive to the change in input size, as complex models generally need more data to be trained effectively.

Results in Table 4 shows model performance when only the first few rows are used for training. We can observe that when the size of the input table is $\le 3$ rows, TabNet outperforms both TBResNet and Table-BERT. This confirms that the less complex model TabNet is not as sensitive to input size compared to TBResNet and Table-BERT.

**Fig. 10:**
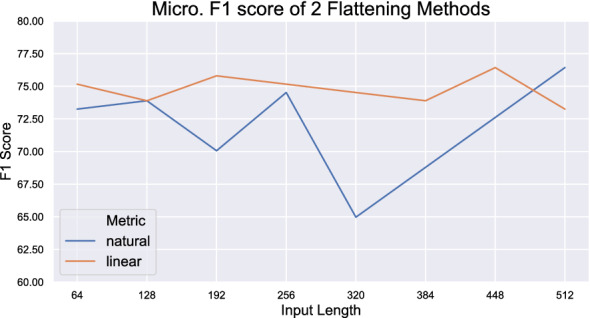
Effect of input length on classification performance of Table-BERT

**Fig. 11:**
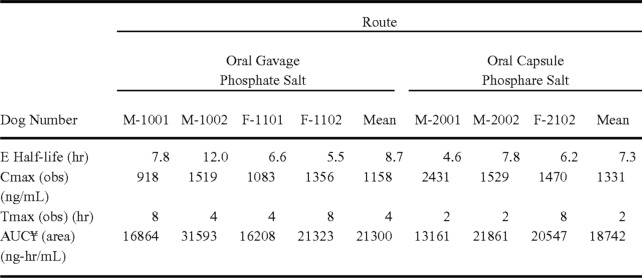
Example of PHARM table which is predicted as OTHER by both Table-BERT and TBResNet. *(US20150259353A1 TABLE 6)*

#### Table-BERT

The maximum length of input for Table-BERT is 512 sub-word tokens. However, the number of words within a table can easily exceed this length limit. Therefore, we also explore the effect of input length on classification performance. As shown in Fig. 10, Table-BERT using Naturalization and Linearization strategies achieves its best performance in all 3 metrics when input size is limited to 448 and 512, respectively (achieving the same performance value).

### Effect of flattening strategy

The complexity in table structure makes it difficult to accurately determine the header rows and columns for chemical patent tables. Different from web tables, patent tables can be organized in much more complex hierarchical structure than web tables. For example, the table with id tabl0008-en in EP2918590A1 has 2 $\texttt {<thead>}$ elements, in which the first one contains a figure that can’t be converted to text solely using the XML file. There are also 3 tables with 8 $\texttt {<thead>}$ elements. Such complex table headers are not trivial to resolve by simple heuristics. On the other hand, some patent tables only have very simple headers (or even no header when the table is used for purely enumerating examples). In fact,  10% of the tables in ChemTables do not have $\texttt {<thead>}$ element while another  20% only have the caption (e.g. “Table 5”) in $\texttt {<thead>}$ element.

For the naturalization approach, our goal is to convert a table into a more human readable format, which is closer to BERT’s input data format. However, when the headers cannot be identified correctly, incorporating wrong headers can introduce extra noise in the input, having a negative effect on classification performance.

As shown in Fig. 10, the Linearization approach outperforms the Naturalization approach when input size is less than 448 sub-words. This is different from what has been observed elsewhere in more generic datasets, where naturalization is more accurate. However, at maximum input length, the Naturalization approach archived its best performance, which is the same as the optimal performance of the Linearization approach. This was expected, as the naturalization approach increased the length of tables by incorporating header information into every cell. By observing the trend of performance change, we find that the naturalization approach could potentially archive better performance if the maximum input length of BERT models were to be further expanded beyond their current limit.

### Error analysis

In this section, we analyze the patterns of errors of different models using the confusion matrix of each category. For most classes, we can observe that large portions (> 50%) of confusions occur w.r.t. the label OTHER. This is because OTHER is only assigned when there is no other label within or beyond Reaxys scheme or guidelines matching the content of the table. Hence, the content of tables labelled as OTHER can contain content which overlaps partially with that of other labels, but not sufficiently to qualify as one of them. For label SPECT|PHYS, most confusions happen between with its two components SPECT and PHYS as tables with label SPECT|PHYS have data of both types, but the proportion of these two types of contents is not necessarily balanced.

We also find that there is a considerably large amount of PHARM tables being classified as OTHER. Especially in the case of the latter, such mismatches are due to the consideration (or lack thereof) of the context in the annotated samples. Indeed, the information provided directly in the table or its attached subsets is often somewhat cryptic and requires additional explanations provided by the surrounding text paragraphs and are thus disregarded by Table-BERT and TBResNet. The label OTHER has moreover a very low inner-annotator agreement score, showing that human experts are also struggling using this label to annotate tables. Hence it is not surprising that most of the confusions produced by our model are between OTHER and the rest of the label set.

The table in Fig. 11 is a typical example: Whilst the table already lists the required species, chemical compound and effect, the text in the paragraph above the table clearly outlines that the experiment evolves around pharmacokinetics.

## Conclusion and future work

In this work, we have presented a new dataset ChemTables of tables extracted from chemical patents, with gold standard annotations on the semantic type of tables, which enables research on applications of deep learning to the task of classifying tables based on their semantics. We also established strong baselines on this dataset with various machine learning methods, including non-neural methods and 3 neural models: TabNet, TBResNet and Table-BERT. The experimental results show that all neural models outperform non-neural baselines in overall performance and categories with more than 100 instances while non-neural baselines perform better on under-represented categories. Our work thus indicates that machine learning models trained on the ChemTables dataset can help in identifying tables of interest to researchers automatically, hence reducing the amount of human effort needed in chemical patent excerption. Furthermore, this dataset can also be used for table representation learning, such as training unsupervised language models like BERT.

The best performing model Table-BERT achieves a performance of nearly 76 micro-averaged $F_1$ score across all classes and shows a significant advantage on relatively under-represented classes in the dataset. The 2-dimensional models show competitive performance given that they have not been pre-trained on any data other than the ChemTables dataset itself. The error analysis showed that even the best performing neural models still fail to tell whether the pharmacological data in tables is relevant or not due to the lack of information from the surrounding text in the model input. Therefore, to solve this problem, it is also important to develop models which learn from both the table and its context. Although the image classification models can learn local features from the convolutional layers, the relationships between cells (e.g. a row header that defines the data type of subsequent cells) are not captured. Hence, it may be interesting to combine sequential models with CNNs to mitigate this problem. The advantage of pre-trained language models observed in this task also motivates us to test their incorporation into models taking 2-dimensional data as inputs, such as TabNet and TBResNet, in future work.

## Footnotes

1 Reaxys^®^ Copyright ©2020 Elsevier Limited except certain content provided by third parties. Reaxys is a trademark of Elsevier Limited. https://www.reaxys.com

2 http://webdatacommons.org/webtables/.

3 https://github.com/zenanz/ChemTables.

4 https://github.com/BlueObelisk/oscar4.

5 List of all patents from which at least one table has been drawn can be found in our GitHub repository https://github.com/zenanz/ChemTables/blob/master/ChemTables_Patent_IDs.txt.

6 ChemTables Dataset http://doi.org/10.17632/g7tjh7tbrj.3
